# Assessing the preparedness of foundation year 1 (FY1) doctors during the transition from medical school to the foundation training programme

**DOI:** 10.1186/s12909-020-01999-5

**Published:** 2020-04-07

**Authors:** Athena Michaelides, Melina Mahr, Gaurav Pydisetty, Jerocin Vishani Loyala

**Affiliations:** University of Nicosia Medical School, Makedonitissis 46, 2417 Nicosia, Cyprus

**Keywords:** Foundation doctor, Junior doctor, Transition, Medical school, Foundation training programme, Preparedness

## Abstract

**Background:**

To assess how ready current FY1 doctors felt when starting their first posting as newly qualified doctors.

**Methods:**

We created an online survey where 45 FY1 doctors in South Yorkshire rated how confident they were across different domains (Induction and Assistantship, Hospital Systems, and Skills) using a scale ranging from 0 (no confidence performing task) to 5 (highly confident).

**Results:**

The *ICE* system and observations had a mean rating of 3.645 (95% CI 3.446 to 3.844), and 4.425 (95% CI 4.248 to 4.602) with a standard deviation (SD) of 1.10 and 0.83 respectively. The radiological platform, referral system, telephone system, and hospital notes had a mean score of 3.149 (95% CI 2.876 to 3.422), 2.340 (95% CI 2.096 to 2.584), 3.573 (95% CI 3.407 to 3.739) and 4.061 (95% CI 3.931 to 4.191), with a SD of 1.281, 1.153, 0.961 and 0.752 respectively. Patient needs and diagnostic procedures had a mean score of 3.933 (95% CI 3.749 to 4.117) and 4.250 (95% CI 4.140 to 4.360) with a SD of 1.190 and 0.944 respectively. Patient care, prescribing, and therapeutic procedures gave a mean score of 3.634 (95% CI 3.432 to 3.836), 4.130 (95% CI 3.959 to 4.301), 3.386 (95% CI 3.228 to 3.544) with a SD of 1.133, 0.958 and 1.256 respectively. No statistically significant difference in the overall mean score was found between respondents who attended medical schools in Yorkshire, and those who had not.

**Conclusion:**

Overall, confidence differs across different domains and categories. A larger sample size across a wider geographical area would allow us to compare and contrast results across the different regions in the UK.

## Strengths and limitations

1. The timing of our survey assesses, as accurately as possible, the transition period from medical school to foundation training - the FY1 cohort started their first foundation training jobs in August and our survey was sent to them in September.
2. The short length of our survey (average time to completion being 5 min) allowed respondents to fully answer the questions in a single sitting with minimal distraction.
3. Analysis of individual questions allowed us to determine which single skill contributed to skewing the mean of a respective category.
4. Our results give an impression of the experiences of FY1 doctors within a single region only since our sample was limited to South Yorkshire.
5. A bigger sample size would render more accurate results, so for the future, we would consider gathering data from the FY1 cohort of other regions to compare and contrast confidence scores across the United Kingdom.

## Article summary

1. This study investigated the confidence level of newly qualified Foundation Year 1 (FY1) doctors for various skills they are expected to perform during their Foundation Training Year.
2. Confidence was assessed by allocating a score of 0–5 to different skills across domains, namely Induction and Assistantship, Hospital Systems, and Skills.
3. We also had an open-ended section which allowed for further explanation of their individual experiences and recommendations for the future.
4. A limitation of the study is that the sample size was small and limited to one region in the UK.
5. The survey was sent out approximately one month following the start of their training which ensured that results are a reflection of experiences during the graduate’s job in the first month, rather than anticipation of the job.

## Background

The transition from medical student to junior doctor represents a crucial stage in the progression of a young doctor’s career. Research on preparedness confirms that this transition can be a stressful experience [1–3]. Junior doctors are a vital part of the medical team responsible for managing the care of patients on a day-to-day basis. This role comprises of responsibilities ranging in number and complexity that junior doctors are expected to be competent in from their first posting [4]. It has been identified that upon entering the Foundation Training Programme as a Foundation Year 1 (FY1) doctor, junior doctors lack confidence or are unfamiliar with certain tasks expected of them. Some known areas of under-preparedness are safe prescribing, clinical reasoning and diagnosis, and early management of emergency patients [4, 5].

Medical schools are tasked with preparing their graduates to be successful doctors. In 2005, 58% of graduates agreed that medical school prepared them well for their first posting. Furthermore, when the medical graduates of 2008 and 2009 were surveyed towards the end of their FY1 posting, only 51% agreed with this statement retrospectively [6, 7]. In 2012, 70% of the graduates agreed that medical schools had prepared them adequately. Two years later, 69.9% of newly qualified doctors agreed or strongly agreed that they were adequately prepared for their first posting [7]. In addition to medical schools’ preparing medical students for the transition to FY1; assistantships, induction, and shadowing also play a crucial role in this transition.

Medical schools are free to design their own teaching programmes as long as they adhere to the outcomes and five themes outlined by the General Medical Council (GMC) [8, 9]. This ensures that their graduates will work safely, promote good practice, and be able to perform the tasks outlined by Tomorrow’s Doctors [10]. UK medical schools can adopt one of three teaching styles: traditional, reformed, or problem-based learning (PBL). The traditional curriculum has a clear pre-clinical and clinical division with discipline-based learning, while PBL is more student-led and self-directed [10, 11]. The reformed teaching style is a combination of core integrated curriculum and PBL [11]. It is unclear and debatable which teaching technique is favorable as they both have advantages and drawbacks. Some universities, such as Oxford University, have a clear distinction between pre-clinical and clinical years, whereas others combine both, easing the transition from theory to practice, like Cardiff University [12, 13]. Norwich Medical School adopted a PBL style curriculum coupled with clinical placements, starting in year one with primary and secondary care. The University of Liverpool and Manchester Medical School have also changed their curriculum to PBL style [11]. There is some evidence that graduates of different medical schools are prepared more than others, however, when comparing all three curriculum types, graduates expressed the same areas of preparedness and weaknesses/concerns [10, 11].

In 2009, the General Medical Council (GMC) updated their medical education guidance entitled ‘Tomorrow’s Doctors 2009’, in which they recommended implementing a new placement for medical students to complete upon finishing medical school and before entering the foundation programme [14]. In this new placement, now termed ‘student assistantship’, students would gradually take on the responsibilities of a Foundation Doctor - a trait that was lacking from medical school placements [15]. With this assistantship in place, the aim was to better prepare newly qualified doctors for the responsibilities, scheduling, and workload of a Foundation Doctor. Because assistantships are organized by the medical school, they are carried out within the institution’s geographical region and not where the students will have their first posting.

A longitudinal questionnaire conducted in March 2019 showed an overall positive correlation between assistantships and level of preparedness, in addition to decreasing anxiety for the first posting as a Foundation Doctor [16]. Due to limited publications on assistantships, additional research is required to assess this relationship further.

Compulsory workplace induction was added to the Foundation Programme in 2012 in the hopes of easing the transition for graduates [17]. This is a workplace-specific process that should cover the employer’s local practices and policies, both clinical and non-clinical. The British Medical Association's ‘Induction for junior doctors 2018’ has a list of key features that should be included in a quality induction programme [18]. Some of these key features include IT and communication arrangements, a clear outline of the job and its requirements, health and safety (fire safety, manual handling and infection control), as well as arrangements made to get paid, go on holiday, organize staff rotas, and take study leave.

As of early 2012, new graduates are all required to shadow an FY1 job a minimum of 4 days prior to starting their posting [4]. This aims to familiarize doctors with the upcoming jobs they will be commencing and develop a relationship with their future colleagues as well as their clinical and educational supervisors [17, 19–22].

As final year medical students currently based at teaching hospitals in the UK, and interested in working for the NHS, we aimed to assess how confident recent graduates felt performing the tasks outlined by the GMC. We have reported junior doctors’ opinions of their own level of confidence upon entering the medical system as FY1 doctors. A quantitative survey was sent approximately one month into their first training post. This survey aimed to assess whether further improvements are needed prior to FY1 training to help the junior doctors’ transition in the future.

## Methods

An online survey was sent to the FY1 doctors placed at hospitals in the South Yorkshire region of the United Kingdom. This included hospitals in Sheffield, Doncaster, Rotherham, and Barnsley. We assessed whether the newly qualified doctors working in these areas graduated from medical schools in the Yorkshire area or beyond. We then assessed whether their medical schools offered an assistantship, as well as whether the hospital they are currently working at offered an induction day/week prior to starting work as an FY1 doctor. The survey was conducted anonymously.

Before sending the survey out to our target population, we conducted a pilot study to ensure we had highlighted the major aspects that define the role of an FY1 doctor. This included the distribution of our draft survey to the local medical education manager, consultant doctors involved in education, and a small number of foundation doctors (FY1s and FY2s). The librarian facilitated the survey design process so that questions were structured, clear, and coherent. This helped optimize the time we were requesting of the FY1s to fill out our survey. As hospitals at different sites may use different systems, we aimed to keep questions general, providing examples in parenthesis should the respondent need clarification.

The competencies of interest to us were those outlined by the General Medical Council (GMC) in the updated version of ‘Tomorrow’s Doctors’ [14]. This document describes the outcomes and standards for undergraduate medical education. For our study, we focused on the specifications that fall part of patient needs, diagnostic procedures, patient care, prescribing, and therapeutic procedures [8, 14]. We also assessed their confidence using different hospital systems such as online patient records, observation charts, radiological platforms, referral systems, using the telephone, and handling patient notes.

The FY1 doctors were asked to rate their level of confidence within each domain according to a 6-point Likert scale: 5 = highly confident (the doctor never doubts his/her ability); 4 = mostly confident (the doctor has occasional doubts); 3 = uncertain (doctor doubts him/herself more often than not); 2 = below adequate (the doctor always doubts him/herself but performs the task anyway); 1 = poor (the doctor avoids performing the task due to self-doubt); and 0 = not at all (the doctor feels he/she cannot perform this task at all). The 6-point scale was chosen as a higher point scale has greater reliability than a 5- or 4-point scale. This gave respondents more options to choose from and limited the ambiguity of each individual rating [23].

We pooled the results together according to the task and confidence level assigned to it. Averages could then be calculated and the difference in mean ratings across the domains presented as a point for further investigation. Across the domains explored, Cronbach alpha is the statistical test we applied to measure the internal consistency of our results. This tested the correlation between different questions of the same category as this is a way to gauge whether similar questions in the survey produce similar mean scores [24, 25]. We used the widely accepted interpretation of the alpha coefficient where ɑ ≥ 0.7 is acceptable; 0.5 < ɑ < 0.7 is deemed questionable; and ɑ ≤ 0.5 is unacceptable [26].

A further analysis was carried out to compare confidence ratings of FY1s who attended medical schools in Yorkshire with those who had not. The premise of using the Welch t-test (unequal variances t-test) to compare non-Yorkshire medical students to the Yorkshire medical students’ confidence ratings is to prove whether the results were significant or if they occurred by chance. The student t-test could not be used as the populations compared did not have equal variances nor equal means [27, 28]. When using the Welch t-test, we have made an assumption that our sample is a reasonable size and normally distributed (bell-shaped distribution curve when plotted on a graph) population [27]. This is the rationale for using an alpha level of 0.05 for this test.

## Results

### Assistantship & Induction

There was an estimate of 172 FY1 doctors in South Yorkshire for 2019 – 102 in Sheffield, 27 in Doncaster, 22 in Rotherham, and 21 in Barnsley. This estimate included doctors in the 2-year Foundation Training Programme, locum, trust grade, and other FY1s and was obtained through contacting each Trust. This estimate did not take into account unexpected changes that occur throughout the year due to supervisor issues, service reconfiguration, and training issues. Of the total 47 respondents, 95.7% (*n* = 45) attended medical schools in the UK prior to entering the Foundation Programme. For this report, we excluded respondents who attended medical schools outside of the UK. Amongst those who attended UK medical schools, 51.1% (*n* = 23) of these medical schools were in the Yorkshire area. An assistantship, defined as a clinical placement in which the medical student assisted and performed the duties of a junior doctor for a few weeks prior to graduating, was offered by 91.1% (*n* = 41) of the respondents’ medical schools. Amongst these, 79.1% (*n* = 34) found it useful to their current FY1 posting.

**Fig. 1:**
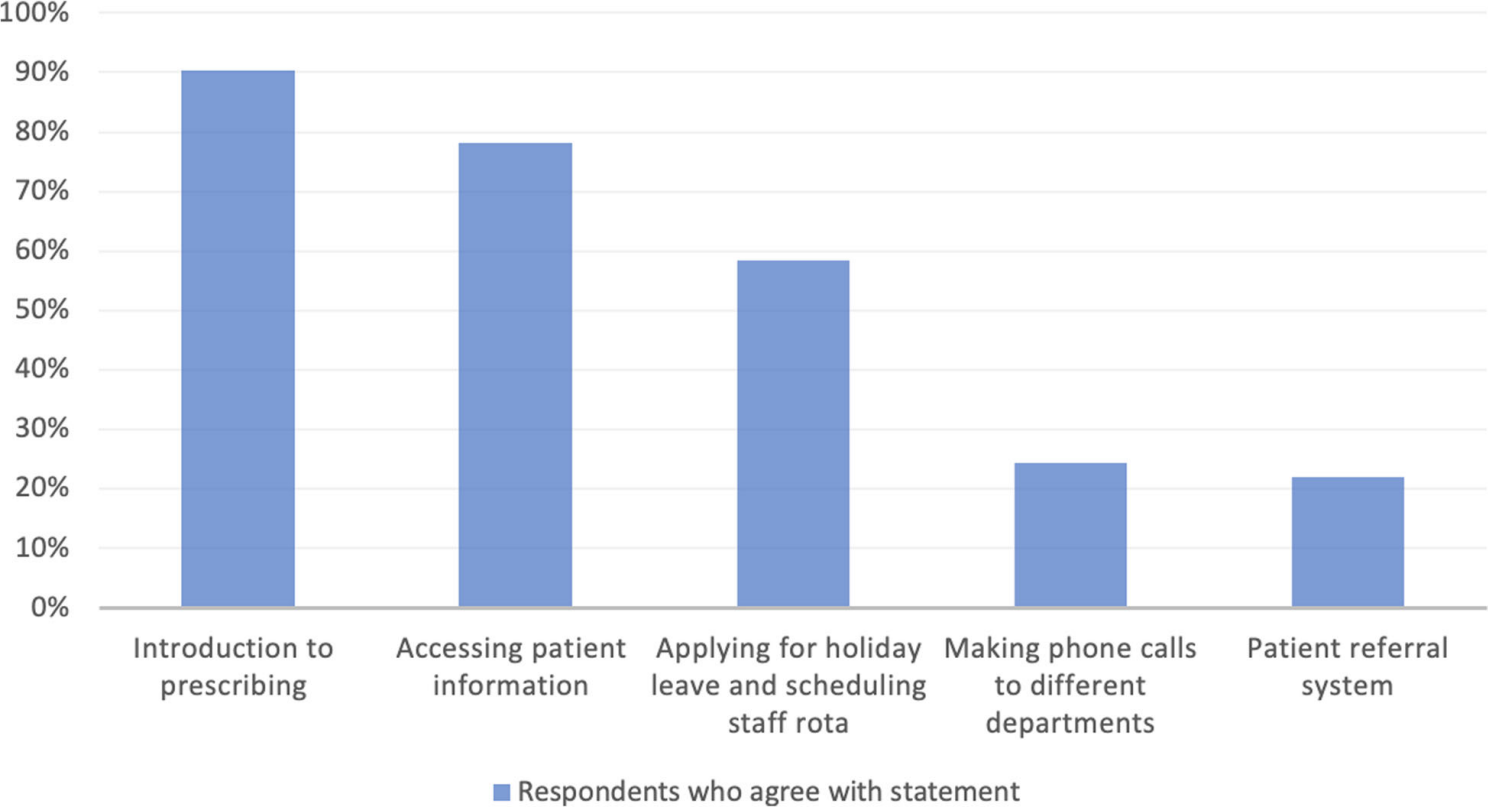
Percentage of respondents who agreed that certain topics were covered during the induction (*n* = 41)

An induction day/week was offered by 100% (*n* = 45) of the hospitals but only 86.7% (*n* = 39) found it useful. When asked details about what induction covered, 41 responses were recorded: 90.2% (*n* = 37) said that induction covered an introduction to prescribing; 78.1% (*n* = 32) agreed that training on how to access patient information such as discharge letters, blood results, and scans was provided; 58.5% (*n* = 24) said that information on applying for holiday leave and scheduling the staff rota was provided; 24.4% (*n* = 10) said that information on how to make phone calls to different departments was provided; and 22.0% (*n* = 9) said that the patient referral system was explained. Figure 1 shows a graphical representation of which topics respondents felt were covered during induction.

### Hospital Systems

The Hospital Systems domain was divided into six categories: online patient records; observation charts; radiological platform; referral system; using the telephone; and paperback patient notes. Each category was investigated in more detail through a series of questions where a confidence rating of 0–5 was allocated for each. A total of 44 responses were recorded for this section. Since each of the six systems had multiple questions that were each given a rating of 0–5, we used *Microsoft Excel* and *IBM SPSS software* to compute the statistics. Table 1 summarizes the results for each Hospital System and the results of theindividual questions that each category was comprised of. The internal consistency of the categories was measured with Cronbach’s alpha coefficient.

**Table 1:** Results of Health Systems analysis as rated by FY1 doctors (sample size = 44) on a scale of 0 (not at all confidence / cannot perform task at all) to 5 (highly confident / never doubts ability)

HEALTH SYSTEM	MEAN	SD	95% CI	α
**Online patient filing system e.g. ICE**	**3.645**	**1.100**	**± 0.199**	**0.750**
*Requesting (bloods, scans,* etc.*)*	3.818	1.040	± 0.316	
*Finding patient records*	3.863	1.025	± 0.312	
*Writing up and sending off discharge letters*	3.166	1.207	± 0.408	
**Observation chart e.g. VitalPac**	**4.425**	**0.830**	**± 0.177**	**0.553**
*Looking up observations*	4.326	1.040	± 0.320	
*Correctly interpreting NEWS2 score*	4.523	0.549	± 0.167	
**Radiological platform e.g. IMPAX**	**3.149**	**1.281**	**± 0.273**	**0.764**
*Looking up patient X-rays, CTs, MRIs* etc.	3.744	1.157	± 0.356	
*Using different viewing features (*i.e. *split screen, changing brightness/contrast* etc.*)*	2.568	1.129	± 0.343	
**Referrals**	**2.340**	**1.153**	**± 0.244**	**0.796**
*Do you know how to use referral system (inpatient, outpatient)?*	2.636	1.203	± 0.366	
*How to refer to a service outside the hospital (community/GP/social services)?*	2.045	1.033	± 0.314	
**Phone system**	**3.573**	**0.961**	**± 0.166**	**0.792**
*Know who to contact*	3.432	0.873	± 0.266	
*Making/taking phone calls*	3.795	0.734	± 0.223	
*Using bleeps*	3.488	1.203	± 0.370	
**Notes**	**4.061**	**0.752**	**± 0.130**	**0.823**
*Clerking patients*	3.955	0.696	± 0.254	
*Note taking in patient file (*i.e. *during ward rounds)*	4.326	0.606	± 0.187	
*Finding patient notes easily in their file*	3.909	0.741	± 0.225	

*Abbreviations*: *FY1* Foundation Year 1, *SD* Standard Deviation, *CI* Confidence Interval, *α* Cronbach’s alpha, *ICE* Integrated Clinical Environment, *NEWS2* National Early Warning Score, *CT* Computed Tomography, *MRI* Magnetic Resonance Imaging, *GP* General Practitioner

We first investigated the online patient filing system. An example of this is the *Integrated Clinical Environment (ICE)* software system which views radiology, pathology, and microbiology results held by the hospital, and allows the laboratory team to look at the information too. It also acts as a platform where certain investigations are requested from the various departments, and discharge letters are written up and sent to the appropriate recipient [29]. On the scale of 0–5, the mean overall rating was 3.645 (95% CI 3.446 to 3.844) with a standard deviation of 1.1. Cronbach’s alpha for this category which was made up of 3 questions was 0.750.

The second aspect that we investigated was confidence in reading and understanding observation charts. Questions focused on the doctor’s ability to look up observation readings and understand National Early Warning Score (NEWS) 2 scores [30]. *VitalPac* is an example of the mobile software used to record patients’ vital signs to track progress and identify acute changes [31]. The mean overall rating for this section was 4.425 (95% CI 4.248 to 4.602) with a standard deviation of 0.830. Cronbach’s alpha for this category which was made up of 2 questions was 0.553.

The next hospital system we looked at was the digital radiological platform. Questions focused on the ability to look up patient x-rays, CTs, and MRIs, and whether or not the doctor felt capable of using the different viewing features for each. An example of a radiological platform employed by hospitals is *IMPAX* – a Picture Archiving and Communication System (PACS) client for a Windows operating system [32]. The mean score was 3.149 (95% CI 2.876 to 3.422) with a standard deviation of 1.281. Cronbach’s alpha for this category which was made up of 2 questions was 0.764.

The referral system was investigated through questions that assessed the doctor's ability to use inpatient and outpatient referral systems, as well as referring to a service outside of the hospital such as the community, general practitioner (GP), or social services. The mean score out of 5 for this section was 2.340 (95% CI 2.096 to 2.584) and standard deviation of 1.153. Cronbach’s alpha for this category which was made up of 2 questions was 0.796.

The telephone system was assessed through questioning whether doctors know who the appropriate person or department is to contact in a given situation, how confident they are making/taking phone calls to/from the various departments, and if they are able to use bleeps (pagers given to junior doctors to respond to situations when requested) effectively. The mean score was 3.573 (95% CI 3.407 to 3.739) with a standard deviation of 0.961. Cronbach’s alpha for this category which was made up of 3 questions was 0.792.

The final system that we looked at was the paper or hardcopy version of hospital notes. Questions focused on confidence clerking patients, note-taking during ward rounds, and the ability to find notes easily within the patient’s file. The mean score was 4.061 (95% CI 3.931 to 4.191) with a standard deviation of 0.752. Cronbach’s alpha for this category which was made up of 3 questions was 0.823.

When asked whether or not the FY1’s would have liked more training on the hospital systems prior to starting the training programme, the responses varied: 38.5% (*n* = 17) expressed a need for more training on online patient filing systems such as *ICE*; only 15.9% (*n* = 7) would have liked more training on observation charts such as Vital Pac; 63.6% (*n* = 28) said that more training on radiological platforms such as *IMPAX* would have been useful; 84.09% (*n* = 37) would have liked training on the hospitals referral system; 43.2% (*n* = 19) would have liked more training on the hospitals phone system; and 26.2% (*n* = 11) on the hard-copy notes and filing systems.

The responses for the Hospital System domain were also analyzed separately for the respondents who attended a medical school in the Yorkshire region, and those who attended medical schools elsewhere. For FY1s who attended medical schools in the Yorkshire area, the overall mean was 3.621 (95% CI 2.367 to 4.875) with a standard deviation of 1.25 compared to a mean of 3.488 (95% CI 2.425 to 4.551) with a standard deviation of 1.063 for those who did not attend a medical school in Yorkshire. Using the t-test, a comparison of non-Yorkshire medical students and the local students’ confidence ratings could prove whether we had a significant result. A t-test value of 0.748 was generated from the mean of the pooled categories.

### Skills

For this section, we focused on the competencies outlined by the GMC in ‘Tomorrow’s Doctors” [14]. The five categories we focused on were Patient Needs, Diagnostic Procedures, Patient Care, Prescribing, and Therapeutic Procedures. A total of 41 responses were recorded for this section. Similar to the Hospital System analysis, each of the five categories had multiple questions that were each given a rating of 0–5. Cronbach’s alpha was used to test internal consistency. Table 2 summarizes the results of the five categories, the results of each question that the categories were comprised of, and Cronbach’s alpha for each category.

**Table 2:** Results of Skills analysis as rated by FY1 doctors (sample size = 41) on a scale of 0 (not at all confident / cannot perform task at all) to 5 (highly confident / never doubts ability)

Skill^**a**^	Mean	SD	95% CI	α
**Patient Needs**	**3.933**	**1.190**	**± 0.184**	**0.158**
*Take observations and record appropriately (temp, resp rate, O*_*2*_*sat, urine output)*	4.682	4.682	± 0.165	
*Carry out peak expiratory flow respiratory function test*	4.634	0.536	± 0.169	
*Ophthalmoscopy*	2.854	1.333	± 0.421	
*Otoscopy*	3.561	1.026	± 0.324	
**Diagnostic Procedures**	**4.250**	**0.944**	**± 0.110**	**−0.899**
*Take blood cultures*	4.341	0.728	± 0.230	
*ABGs*	4.049	0.805	± 0.254	
*Venepuncture*	4.610	0.542	± 0.171	
*Measure capillary blood glucose*	4.195	1.030	± 0.325	
*Urine dipstick*	4.707	0.461	± 0.145	
*Carry out 3 and 12 lead ECG*	4.244	1.044	± 0.329	
*Take and/or instruct patient how to take a swab*	3.610	1.321	± 0.417	
**Patient Care**	**3.634**	**1.133**	**± 0.202**	**0.540**
*Perform surgical scrubbing up*	3.927	0.959	± 0.303	
*Preparation of IV infusion*	3.415	1.264	± 0.399	
*Use correct techniques for moving and handling patients (including frail)*	3.561	1.119	± 0.353	
**Prescribing**	**4.130**	**0.958**	**± 0.171**	**0.778**
*Instruct patients in the use of devices for inhaled medication*	4.244	0.800	± 0.252	
*Prescribe and administer oxygen*	4.293	0.782	± 0.247	
*Prepare and administer injectable (IM, SC, IV) drugs*	3.854	1.195	± 0.377	
**Therapeutic Procedures**	**3.386**	**1.256**	**± 0.158**	**−0.837**
*IV cannulation*	4.317	0.820	± 0.259	
*Carry out safe & appropriate blood transfusion*	3.585	1.095	± 0.346	
*Male & female catheterisation*	3.415	0.865	± 0.273	
*Carry out wound care and basic wound closure and dressing*	3.073	1.292	± 0.408	
*Carry out NG tube placement*	2.683	1.491	± 0.470	
*Use local anaesthetics*	3.244	1.261	± 0.398	

*Abbreviations*: *FY1* Foundation Year 1, *SD* Standard Deviation, *CI* Confidence Interval, ***α*** Cronbach’s alpha, *temp* Temperature, *resp* Respiratory, *sat* Saturation, *ABGs* Arterial blood gases, *IV* Intravenous, *IM* Intramuscular, *SC* Subcutaneous, *NG* Nasogastric

^a^Skills derived from General Medical Council. (2009). *‘Tomorrow’s Doctors*. ‘Recommendations on undergraduate medical education’

Patient Needs were assessed by asking doctors to assess their ability to carry out the following: taking observations such as temperature, respiratory rate, oxygen saturation, and urine output; carrying out peak expiratory flow and respiratory function tests; performing ophthalmoscopy; and performing otoscopy. Out of 5, a mean score of 3.933 (95% CI 3.749 to 4.117) was recorded with a standard deviation of 1.190. Cronbach’s alpha for this category which was made up of 4 questions was 0.158.

The Diagnostic Procedures that we investigated included taking blood cultures, performing venepuncture, taking arterial blood gases (ABGs), measuring capillary blood glucose, performing urine dipstick, carrying out a 3 and 12 lead electrocardiogram (ECG), and taking and instructing a patient how to take a swab. The mean score was 4.250 (95% CI 4.140 to 4.360) with a standard deviation of 0.944. Cronbach’s alpha for this category which was made up of 7 questions was -0.899.

For the Patient Care category, we investigated confidence in tasks such as surgical scrubbing up, preparation of intravenous (IV) infusions, and using correct techniques for moving and handling patients. The mean score was 3.634 (95% CI 3.432 to 3.836) with a standard deviation of 1.133. Cronbach’s alpha for this category which was made up of 3 questions was 0.540.

The Prescribing category assessed the doctors’ ability to correctly instruct patients on the use of devices for inhaled medication, prescribing and administering oxygen, and preparing and administering injectable (intramuscular (IM), IV, and subcutaneous (SC)) drugs. The mean score was 4.130 (95% CI 3.959 to 4.301) with a standard deviation of 0.958. Cronbach’s alpha for this category which was made up of 3 questions was 0.778.

The final category focused on Therapeutic Procedures. This included IV cannulation, carrying out safe and appropriate blood transfusion, male and female urinary catheterization, carrying out wound care and basic wound closure and dressing, use of local anesthetics, and correctly inserting a nasogastric (NG) tube. The mean score for this section was 3.386 (95% CI 3.228 to 3.544) with a standard deviation of 1.256. Cronbach’s alpha for this category which was made up of 6 questions was -0.837.

Further to this, the responses for the Skills domain were analyzed separately for respondents who attended medical schools in the Yorkshire region, and those who attended medical schools elsewhere. For FY1s who attended medical schools in the Yorkshire area, the overall mean was 3.687 (95% CI 2.265 to 5.109) with a standard deviation of 1.422 compared to a mean of 4.058 (95% CI 3.206 to 4.910) with a standard deviation of 0.852 for those who did not attend a medical school in Yorkshire. The t-test was used again to compare non-Yorkshire medical students and the local students’ confidence ratings to determine whether we had a significant result. A t-test value of 0.151 was generated from the mean of the pooled categories.

## Discussion

### Assistantship & Induction

The assistantship, which has recently been introduced, has proven to be beneficial in the transition from medical school to the Foundation Training Programme. Of all the responses, 90.2% of participants agreed that induction covered an introduction to prescribing, while only 22.0% agreed that the patient referral system was explained. This shows a discrepancy in the topics thought to be covered during induction.

Most of the free-text responses in the general recommendations section were about induction and assistantship. Respondents felt that the assistantship would have been more beneficial if they had been offered the opportunity to undertake an assistantship outside of their medical school region. This would have allowed them to familiarize themselves with a system more representative of where they would be working as FY1s. Other recommendations included a general plea for more responsibility as a final year medical student, and more time spent shadowing on the ward and recapping practical skills. Another suggestion emphasized the desire for medical students to be encouraged to be autonomous in suggesting patients’ management plans, and later have this checked by a senior doctor rather than undertaking a passive observational role. This finding is consistent with a study done in 2010 where31 junior doctors also expressed that more responsibilities during placements and shadowing would be beneficial [33].

Overall, respondents found inductions and assistantships useful although stated that proper instruction on how to use the e-portfolio and e-learning systems would have been beneficial, as well as an explanation of the requirements for the Annual Review of Competence Progression (ARCP). Others suggested the following: guidance on where information is kept on the intranet and how to access guidelines; the provision of a clinical session that explains how the medical equipment works at the hospital where the doctor will be placed since equipment differs across the trusts; and more emphasis on teaching them about trust procedures and protocols.

### Hospital systems

In the section assessing Hospital Systems, the highest confidence score was achieved for reading and understanding observation charts (mean 4.425). This corresponds to the low 15.9% of respondents who stated that more training would have been useful before starting the foundation training programme.

The lowest score for this section was for using the hospital referral system (mean 2.340). This low confidence score corresponds to the lack of training provided during induction - only 22% of respondents said that the hospital referral system was explained. Further to this, 84% of respondents expressed that more training on referrals would have been useful before starting the training programme.

Another hospital system that is worth mentioning is the radiological platform: this achieved the second-lowest score (mean 3.149) after the hospital referral system. A total of 63.6% of respondents said that more training would have been useful before startingthe training programme. To improve these scores in the future, further education and training around hospital referrals and radiological platforms could be provided during the induction day/week.

Cronbach alpha identified whether the items pooled within each category of the domain were closely related to each other. Online patient filing system, radiological platform, referrals, phone systems, and patient notes yielded a Cronbach alpha score of above 0.7. This value re-iterates the internal consistency of the results showing that items within these categories are comparable with each other. The Observation Chart category score is between 0.5 to 0.7, which deems to be questionable – this can be caused by the lack of items asked in this area and a difference in covariance between the two questions.

Since medical students attend clinical placements at hospitals and clinics in the area of their medical schools, we hypothesized that the FY1s who attended medical schools in the Yorkshire region would be more confident in their abilities during their first posting at hospitals in this same area. However, this did not prove to be true. There was no statistically significant difference, proving that having a first training post in the region where the FY1 attended medical school does not contribute to a better self-rated level of confidence. This is justified as the calculated t-test value (0.748) is less than the critical t-value (2.228). Although this proved true for our study, we cannot conclude that this is true for all FY1s in the UK due to our small sample size.

### Skills

In the domain assessing doctors’ skills, the category assessing Diagnostic Procedures obtained the highest confidence rating (mean 4.250) while the lowest score was obtained for Therapeutic Procedures (mean 3.386).

A discrepancy was noted amongst the individual items assessed under Therapeutic Procedures: higher scores were recorded for tasks such as IV cannulation (mean 4.317), carrying out blood transfusions (mean 3.585), and inserting male and female urinary catheters (mean 3.415); lower scores were allocated to carrying out wound care and basic closure and dressing (mean 3.073); while the lowest score was for NG tube placement (mean 2.683). The low scores for these individual tasks skewed the final mean score for the Therapeutic Procedures category. For future recommendations and skills improvement, emphasis should be placed on wound care and NG tube placement.

A discrepancy was also noted in the category assessing Patient Needs (mean 3.933): higher scores were obtained for taking and recording observations (mean 4.682) and carrying out peak expiratory flow respiratory function tests (mean 4.634) compared to performing ophthalmoscopy (mean 2.854) and otoscopy (mean 3.561). As ophthalmoscopy and otoscopy are specialized skills, there is minimal focus on teaching these at medical school and junior doctor level.

In the Skills domain, the Prescribing category yielded a Cronbach alpha score of above 0.7 which is in line with an acceptable internal consistency of the items within this category. The Patient Care category lies between 0.5 to 0.7, which deems to be questionable. Patient Needs, Diagnostic Procedures, and Therapeutic Procedures were below 0.5. In the Patient Needs category, ophthalmoscopy and otoscopy achieved a lower score than respiratory function test and recording observations which in turn decreased the alpha value, as the mean values between these four items were not closely related. The categories with an alpha score of 5 or below are attributed to large variations in mean values between items within the category. Negative values of alpha in the Diagnostic and Therapeutic Procedures categories are attributed to data error. Data error can be caused by the small sample size and that the items can have a multi-dimensional meaning. This means that questions are not interpreted in a way that reflects the category the questions are in, and so give a lower or negative alpha value. A method of resolving this would be to remove items within the category that have low-item correlations. However, we did not do this as we chose to stick to the GMCs grouping of items as per the ‘Tomorrow’s Doctors’ document and instead report on the weak association between certain competencies within a category.

Similar to the Hospital Systems domain, there is no statistically significant difference in confidence scores for the Skills domain between FY1s who attended medical schools in Yorkshire, and those who did not. This suggests that medical schools across the UK are equipping students with a similar set of skills to take on an FY1 post in any location with equal self-rated confidence to his/her colleagues. This is supported by the calculated t-test value (0.151) being less than the critical t-test value (2.306).

## Conclusion

Due to this study being a retrospective survey done in September, our results are a reflection of experiences during the graduate’s job in the first month, rather than anticipation of the job. Based on the data at hand, graduates are performing well in their tasks. Since confidence scores vary for different practical skills and abilities to use the hospital systems, there is room for improvement. Because our sample size was limited and only about one-fourth of FY1s responded, the data does not represent the entire FY1 cohort of South Yorkshire, however, it does describe newly graduated doctors’ feedback and recommendations.

As we do not have each of the individual hospitals’ induction programmes, we cannot say for certain what needs to be improved on in each hospital trust. However, this data does provide information on what was useful to the graduates and in which areas they wish to have more training such as the hospital referral system, radiology platform (e.g. *IMPAX*), and hospitals phone system. Overall induction was found useful. The lowest scoring skills, such as wound care and NG tube placement, will require more teaching and support in the future to increase graduate’s confidence in these skills.

Medical student transition to Foundation Year 1 doctor has been vastly researched, and there have been multiple improvements along the years to make the transition as smooth as possible by improving placements in medical school to starting assistantships and implementing paid shadowing during induction. Individuals’ further comments emphasized that graduates wished to have longer assistantships as students or shadowing time as FY1s, as well as the opportunity to do the assistantship in the hospital that they would be starting work as this could potentially decrease the unfamiliarity that junior doctors experience in the first weeks of starting their job. This would be something to implement in the future planning of the assistantship programme and Foundation Programme inductions.

## Supplementary information

**Additional file 1.**

## Data Availability

The datasets used and/or analyzed during the current study are available from the corresponding author upon request.

## Abbreviations

ABG: Arterial blood gas
ARCP: Annual Review of Competence Progression
CI: Confidence Interval
CT: Computer Tomography
ECG: Electrocardiography
FY1: Foundation Year 1
GMC: General Medical Council
GP: General Practitioner
ICE: Integrated Clinical System
IM: Intramuscular
IV: Intravascular
MRI: Magnetic Resonance Imaging
NEWS: National Early Warning Score
NG: Nasogastric
PACS: Picture Archiving and Communication System
PBL: Problem based learning
SC: Subcutaneous
SD: Standard deviation
