# Supplementary material for: Assessing the preparedness of foundation year 1 (FY1) doctors during the transition from medical school to the foundation training programme
Source: BMC Med Educ. 2020 Apr 7;20:106. doi: 10.1186/s12909-020-01999-5 (PMC7137288; doi:10.1186/s12909-020-01999-5)
Supplement: Supplementary file 1 — Additional file 1. [file 12909_2020_1999_MOESM1_ESM.docx]

**SAMPLE SURVEY SENT TO FY1s**

**SECTION A: ASSISTANTSHIP & INDUCTION**

1. Did you study at a medical school in the UK? Yes/No

2. Did you study at a medical school in Yorkshire? Yes/No

3. Did your medical school offer an assistantship (clinical placement in which you assisted & performed the duties of a junior doctor for a few weeks) prior to graduating? Yes/No

If yes please specify region:

4. If yes to above, did you find the assistantship useful for your current F1 posting? Yes/No

5. Did your hospital offer an induction day/week for you? Yes/No

6. If yes to above, did you find it helpful? Yes/No

Other Comments:

7. Did induction cover the following?

| Training on how to access patient information (Discharge letters, Bloods, Vitals, Scans) |  |
| --- | --- |
| How to make phone calls to different departments? |  |
| Patient referrals |  |
| Prescribing (drug card, Lorenzo or other software) |  |
| Applying for holiday leave and scheduling rota |  |

**SECTION B: HOSPITAL SYSTEMS**

Upon entering F1, how confident were you in the following tasks?

5 = Highly confident (I never doubt my ability)

4 = Confident (I have occasional doubts)

3 = Adequate (I doubt myself more often than not)

2 = Below adequate (I always doubt myself but perform the task anyway)

1 = Poor (I avoid performing due to self-doubt)

0 = Not at all (I cannot perform this task at all)

|  | 5 | | 4 | | 3 | 2 | | 1 | 0 | |
| --- | --- | --- | --- | --- | --- | --- | --- | --- | --- | --- |
| **Online patient filing system e.g. ICE** |  | |  | |  |  | |  |  | |
| Requesting bloods/scans |  | |  | |  |  | |  |  | |
| Finding patient records |  | |  | |  |  | |  |  | |
| Writing up and sending off discharge letters |  | |  | |  |  | |  |  | |
| Would you have liked more training on this prior to starting FY1? | YES | | | | | NO | | | | |
| **Observation chart e.g. VitalPac** |  | |  | |  |  | |  |  | |
| Looking up observations |  | |  | |  |  | |  |  | |
| Correctly interpreting NEWS2 scores |  | |  | |  |  | |  |  | |
| Would you have liked more training on this prior to starting FY1? | YES | | | | | NO | | | | |
| **Radiological Platform e.g. IMPAX** |  | |  | |  |  | |  |  | |
| Looking up patient x-rays, CTs, MRIs? |  | |  | |  |  | |  |  | |
| Using different viewing features (i.e. splits screen, changing brightness/contrast etc) |  | |  | |  |  | |  |  | |
| Would you have liked more training on this prior to starting FY1? | YES | | | | | NO | | | | |
| **REFERRALS** |  |  | |  | |  |  | | |  |
| Do you know how to use referral system? (inpatient, outpatient) |  |  | |  | |  |  | | |  |
| How to refer to a service outside the hospital (community/GP/social services) |  |  | |  | |  |  | | |  |
| Would you have liked more training on this prior to starting FY1? | YES | | | | | NO | | | | |
| **PHONE** |  | | | | |  | | | | |
| Know who to contact |  |  | |  | |  |  | | |  |
| Making & taking phone calls (to micro, endoscopy, radiology etc) |  |  | |  | |  |  | | |  |
| Using bleeps |  |  | |  | |  |  | | |  |
| Would you have liked more training on this prior to starting FY1? | YES | | | | | NO | | | | |
| **NOTES** |  | | | | |  | | | | |
| Clerking patients |  |  | |  | |  |  | | |  |
| Note taking in patient file (i.e. during ward rounds) |  |  | |  | |  |  | | |  |
| Find patient notes easily in his/her file |  |  | |  | |  |  | | |  |
| Would you have liked more training on this prior to starting FY1? | YES | | | | | NO | | | | |

**SECTION C: SKILLS**

Please rate how confident you are in your ability to perform the following tasks:

|  | 5 | 4 | 3 | 2 | 1 | 0 |
| --- | --- | --- | --- | --- | --- | --- |
| **PATIENT NEEDS** |  |  |  |  |  |  |
| Take obs and record appropriately (temp, resp rate, O2 sat, urine output) |  |  |  |  |  |  |
| Carry out peak expiratory flow respiratory function test |  |  |  |  |  |  |
| Ophthalmoscopy |  |  |  |  |  |  |
| Otoscopy |  |  |  |  |  |  |
| **DIAGNOSTIC PROCEDURES** |  |  |  |  |  |  |
| Take blood cultures |  |  |  |  |  |  |
| ABGs |  |  |  |  |  |  |
| Venepuncture |  |  |  |  |  |  |
| Measure capillary blood glucose |  |  |  |  |  |  |
| Urine dipstick |  |  |  |  |  |  |
| Carry out 3 and 12 lead ECG |  |  |  |  |  |  |
| Take and/or instruct patient how to take a swab |  |  |  |  |  |  |
| **PATIENT CARE** |  |  |  |  |  |  |
| Perform surgical scrubbing up |  |  |  |  |  |  |
| Set up an IV infusion |  |  |  |  |  |  |
| Use correct techniques for moving and handling patients (including frail) |  |  |  |  |  |  |
| **PRESCRIBING** |  |  |  |  |  |  |
| Instruct patients in the use of devices for inhaled medication |  |  |  |  |  |  |
| Prescribe and administer oxygen |  |  |  |  |  |  |
| Prepare and administer injectable (IM, SC, IV) drugs |  |  |  |  |  |  |
| **THERAPEUTIC PROCEDURES** |  |  |  |  |  |  |
| IV cannulation |  |  |  |  |  |  |
| Carry out safe & appropriate blood transfusion |  |  |  |  |  |  |
| Male & female catheterisation |  |  |  |  |  |  |
| Carry out wound care and basic wound closure and dressing |  |  |  |  |  |  |
| Carry out NG tube placement |  |  |  |  |  |  |
| Use local anaesthetics |  |  |  |  |  |  |

**SECTION D: IMPROVEMENTS & RECOMMENDATIONS**

Any other general recommendations or things that would have made your transition from medical school to FY1 easier?
